# Preferences for Mobile Apps That Aim to Modify Alcohol Use: Thematic Content Analysis of User Reviews

**DOI:** 10.2196/63148

**Published:** 2025-03-19

**Authors:** Megan Kirouac, Christina Gillezeau

**Affiliations:** 1 Center on Alcohol, Substance use, And Addictions, The University of New Mexico, Albuquerque, NM, United States, 1 505-925-2300, 1 505-925-2351

**Keywords:** alcohol mobile app, mHealth, alcohol use disorder, user-centered design, alcohol, user-centered, user, reviews, usefulness, mobile health app, content analysis, drinking, health tool

## Abstract

**Background:**

Nearly one-third of adults in the United States will meet criteria for alcohol use disorder in their lifetime, yet fewer than 10% of individuals who meet for alcohol use disorder criteria will receive treatment for it. Mobile health (mHealth) applications (apps) have been suggested as a potential mechanism for closing this treatment gap, yet there is a wide variety of quality and integrity within these apps, leading to potential harms to users.

**Objectives:**

The aim of this paper is to systematically record and qualitatively examine user reviews or mHealth apps to identify features in the existing apps that may impact usefulness and adoption of them.

**Methods:**

The researchers used Apple App and Google Play stores to identify mHealth apps that were focused on modifying alcohol use and treating common comorbidities. Apps that were free without in-app purchases and provided multiple features for users were included. User reviews from the apps were downloaded and coded using content analysis.

**Results:**

A total of 425 unique apps were found in our search. Of these, the majority of apps (n=301) were excluded from the present analyses for not focusing on reducing alcohol-related concerns (eg, many apps were for purchasing alcohol). Eight apps were identified and had user reviews downloaded. The apps examined in this study were VetChange, SMART, DrinkCoach, SayingWhen, AlcoStat, Celebrate Recovery, TryDry, and Construction Industry Helpline. A total of 370 reviews were downloaded and 1353 phrases were coded from those reviews into a total of 11 codes. The 5 most common themes identified were praise (498 counts coded; 36.831%), tools (150 counts coded; 11.062%), suggestions for improvement (118 counts coded; 8.756%), criticism (105 counts coded; 7.768%), and tracking (104 counts coded; 7.724%).

**Conclusions:**

The current findings suggest that alcohol mobile app users broadly found the apps helpful in reducing their drinking or meeting their drinking goals. Users were able to identify features that they liked or found helpful in the apps, as well as provide concrete feedback about features that they would like included or improved. Specifically, flexible and expansive tracking features and comprehensive whole health tools were cited as valuable and desired. App developers and those looking to expand access to and uptake of alcohol reduction apps may find these user reviews helpful in guiding their app development.

## Introduction

It has recently been estimated that a third of US adults engage in risky alcohol use and just under a third of US adults (29%) will meet criteria for alcohol use disorder (AUD) in their lifetime [1]. Moreover, only 7.9% (2.3 million people aged 12 years and older) of individuals who met criteria for AUD in the United States received any kind of formal treatment [2]. Common reasons for not engaging in treatment include the fear of stigma or privacy concerns and lack of access to in-person resources [3]. Further complicating treatment utilization is the fact that individuals with AUD have a higher prevalence of other co-occurring mental health conditions than individuals with nonsubstance use disorder diagnoses [4]. Rates of co-occurring anxiety, depression, posttraumatic stress disorder (PTSD), or attention-deficit/hyperactivity disorder (ADHD) have been found to be particularly high among individuals diagnosed with AUD [5]. For these individuals with co-occurring diagnoses, formal treatment is often restricted to providers qualified to treat those co-occurring diagnoses simultaneously or to health care systems that are capable of providing coordinated care [4]. Unsurprisingly, having co-occurring AUD and other mental health diagnoses is often associated with poorer treatment outcomes [6].

Mobile apps and other mobile health (mHealth) interventions have been proposed as one potential solution to help overcome many of the current barriers for receiving help with one’s relationship to alcohol [7]. First, mHealth interventions are highly accessible, given that at least 85% of all US adults report owning a smartphone, including members of historically underserved communities [8]. Second, mHealth interventions are also ready 24/7 and available for brief, impromptu interactions compared with scheduled appointments during business hours associated with traditional in-person treatment. Accordingly, mHealth interventions hold promise to overcome access barriers traditional treatments face: geographic and provider restrictions [7].

While some critics argue that mHealth interventions are limited in that clients will not feel connected to a mobile app the same way they would to an in-person provider, some research has shown that clients can develop a therapeutic alliance with their mHealth tools [9,10]. Furthermore, mHealth interventions have been touted as particularly well suited to reduce stigma-related barriers compared with in-person treatments where individuals have to attend treatment visits or clinics where they must interact with others who may judge or stigmatize them [7]. Finally, mHealth interventions allow clients to have levels of autonomy, flexibility, personalization, and depth that is often unparalleled in traditional individual or group treatments [7]. For example, mHealth interventions are often designed with tools and resources sections of the intervention that clients may navigate at their own pace and based on their own individual interests and needs. Instead of having to navigate complex health care systems or potentially inaccurate or unreliable web-based resources, mHealth interventions can be programmed to contain reliable and accurate information that may be relevant for some clients (eg, psychoeducation about PTSD symptoms and PTSD symptom monitoring contained on the National Center for PTSD’s VetChange mobile app for alcohol use).

Several mobile apps have been developed to target alcohol use concerns, yet the quality and integrity of those apps are highly varied. For instance, many alcohol-focused apps are created commercially with limited oversight from government and scientific communities. A recent review highlighted the myriad potential harms of unregulated health-focused apps, including apps that are falsely advertised as “safety apps” but instead “promote binge drinking” [11]. Even apps that are not designed for-profit or that do not contain overtly harmful content are potentially harmful due to the lack of oversight and regulation since they may not be evidence-based and may, therefore, fail to work as desired. Not only do these apps potentially harm users by taking their time to no positive outcome but may also cause secondary harms by making app users feel that there is no help possible for them. As pointed out by Businelle and colleagues [7], “nearly all of the mHealth interventions that are available for download today have not been evaluated for efficacy or effectiveness. Thus, individuals are downloading interventions that may not be effective for addressing their problems, which could delay or discourage the use of future well-studied and effective interventions” [7]. Moreover, recent studies suggest that the utilization of existing mobile apps remains low; yet when apps are used, there is a positive association between user engagement and intervention effectiveness [7]. Accordingly, there is a critical need to evaluate which mobile apps may be evidence-based and which apps are well used in order to inform the public about which apps may be most helpful. One such strategy to accomplish this goal is to examine user reviews of existing apps since user ratings in digital marketplaces have been found to influence app quality and, therefore, impact the usage of that app [7]. Information from these publicly available app user reviews may be used to inform app refinement without associated costs of User Experience (UX) or other User-Centered Design research projects. Therefore, the aim of this paper was to systematically review freely available alcohol mobile apps, identify potentially evidence-based options, and conduct qualitative analyses of user reviews to identify features of existing apps that may impact use and helpfulness of mHealth interventions for alcohol use.

## Methods

### Ethical Considerations

All data used in this study were publicly available as posted by users themselves on Apple App and Google Play app stores. Accordingly, it is within the institutional review board’s policies for the authors’ home institution to consider this research exempt from institutional review board review. Moreover, all data presented in this study were analyzed without usernames and findings herein are presented in aggregate. Where specific quotes are used as examples of thematic codes, no identifying information was included.

### Data Selection Procedures

Both authors for this study have prior qualitative data analytic experience and met at the beginning of the study to discuss procedures for this study aims in February 2024. The first step in achieving the proposed study aims was to identify apps that were potentially evidence-based. The authors searched in the Google Play and Apple App Stores for mHealth apps focused on alcohol. Given the high degree of comorbidity of AUD with other mental health diagnoses, and the stigma associated with AUD specifically, app search terms were extended beyond alcohol alone. Five search terms were used in Google Play and the Apple App Stores: “alcohol,” “alcohol and depression,” “alcohol and anxiety,” “alcohol and PTSD,” and “alcohol and ADHD.” Depression, anxiety, PTSD, and ADHD were selected since those are the mental health diagnoses with the highest prevalence of comorbidity with alcohol [5]. The authors independently reviewed their results to exclude duplicates and restricted the results to apps that were available for free, without in-app purchases, to prevent potentially harmful, for-profit corporate apps from being included in final analyses. Next, authors proposed to exclude apps that were solely for single purposes, such as blood alcohol concentration calculating apps, and any disagreements were resolved through mutual discussion and reaching consensus. Finally, since the current authors are fluent only in English, all non-English apps were excluded from this study. Of the remaining apps, reviews were extracted using the Export Comments website. Since several apps contained only few reviews and other apps contained hundreds of reviews, the authors restricted the number of exported reviews to only the most recent 50 reviews in Google Play and 50 reviews in the Apple App Store. Although this restricted the available data, it allowed results to be more balanced toward the apps generally, rather than app-specific for the most widely reviewed apps. Moreover, reviews limited to the most recent reviews ensured that outdated reviews were not considered in this study, which is particularly important, given that the widely reviewed apps may have been updated such that previous reviews are no longer relevant. App store searches and user review extractions were completed in March 2024.

### Qualitative Analyses

Exported user reviews were downloaded into a spreadsheet program in which coding was conducted. Conventional content analysis was used to examine thematic content to user reviews [12,13], which is a method of qualitative data analysis used to identify themes in text data through systematic classification. In this approach, the researchers examine the data with no preconceived, theory-based hypotheses about what kinds of codes or themes will be found and instead use the data itself to drive the codes identified.

Initial themes were identified by both authors independently generating codes for a third of the reviews from each app identified for data analysis. These codes were then compared by the authors, discussed, and a reviewed codebook was agreed upon. Based on qualitative methodology recommendations and this study aims, the following codebook rules were agreed upon by the authors: (1) assign only 1 code per utterance, with “utterances” distinguished by parts of speech (eg, “and”) or punctuation; (2) emojis are not to be coded, given the propensity for emojis to be transcribed as nonsubstantive text in the data extraction (ie, smiley faces were distorted into uninterpretable characters); (3) specific features identified within coded themes should be noted (eg, when concrete tools or specific tracking features are mentioned); and (4) any time a user review is explicitly not referring to the mobile app itself should be coded as “null” (eg, reference to in-person meetings being helpful vs the app itself). These codebook rules were established to ensure clarity, prevent overlap, and prevent artificially inflating interrater reliability [14,15].

The authors then completed coding using the revised codebook for a different third of the reviews from each app. The selection was made to code a third of reviews at each of these 2 stages based on the authors’ prior experience with qualitative data analysis and expectation for it to take approximately 3 iterations to finalize a codebook. However, the second iteration of the codebook was agreed to be sufficient after the second third of data were independently coded and then discussed by the authors. This final codebook was used to code all of the app user review data by each author until 80% interrater consistency was achieved, which is considered the standard consistency for these types of analyses [16]. Specifically, both authors used an iterative process of coding to create the final codebook and increase rater agreement on the application of each code to each utterance in user reviews. Once the codebook was finalized, raters compared their codes and discrepant codes were flagged and rereviewed by both raters who were then allowed to reconsider their coding upon closer review. Any final discrepancies were discussed by both raters until agreement was achieved and overall interrater consistently met the predetermined 80% guidance from the empirical literature.

## Results

### Apps Analyzed

**Figure 1.**
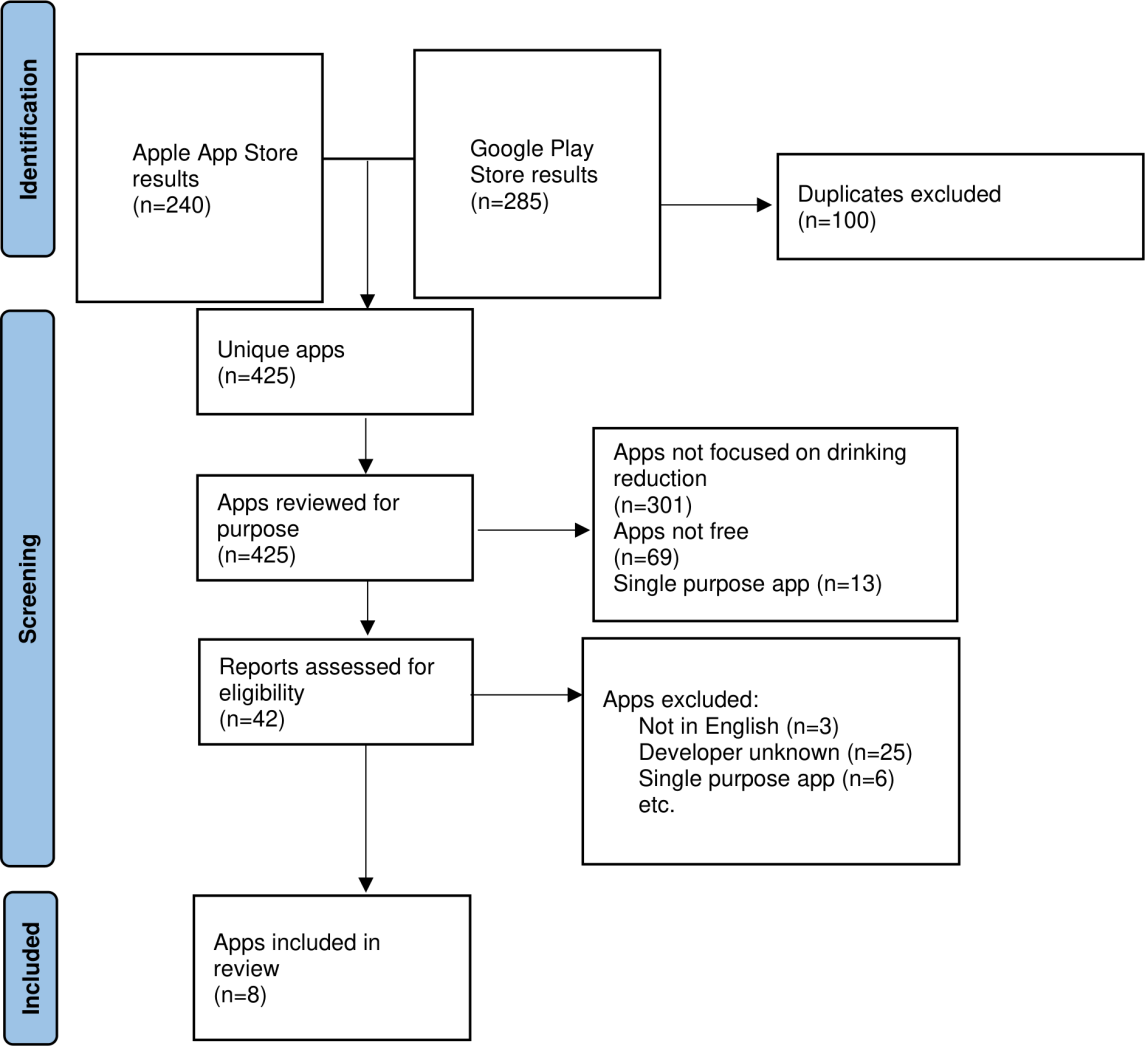
Flow diagram of app store search results and inclusion in the present analyses.

Data extraction was completed in early 2024; see Figure 1 for a flow diagram of app store search results. More than 200 apps were initially returned in search results within Google Play (n=285) and Apple App Stores for the specified search terms (n=240; n=425 unique results between both stores). The majority of these apps were excluded because they were for-profit (ie, had in-app purchases), were not mHealth apps (eg, alcohol-purchasing apps), or were explicitly harmful (ie, alcohol-drinking game apps). The remaining apps were excluded because they were of single purpose (eg, blood alcohol concentration calculators), or were not in English. The remaining results were researched by the authors to determine whether they were made by reputable and legitimate entities (eg, governmental agencies such as the National Center for PTSD) as a proxy for potential evidence basis of the app content. Findings were discussed until a consensus was established for which apps to include. A total of 8 apps were retained for data analysis: VetChange, SMART, DrinkCoach, SayingWhen, AlcoStat, Celebrate Recovery, TryDry, and Construction Industry Helpline. See Table 1 for a list of each of these apps and relevant descriptors. Each app had varying levels of user reviews provided, with Celebrate Recovery and TryDry being the only 2 apps that had more than 100 user reviews on each system platform. Accordingly, these 2 apps’ user review data extraction was limited to only the most recent 50 reviews for Google and Apple (total of 100 reviews for each app). The other apps contained the following number of reviews: VetChange (n=14), SMART (n=35), DrinkCoach (n=51), Saying When (n=56), AlcoStat (n=7), and Construction Industry Helpline (n=7). A total of 370 reviews were included in this study (N=370).

**Table 1.** Name and description of app store search results included in the present analyses.

App name	Number of user reviews	Platform	Developer	Description
AlcoStat	7	iOS	Andrey Shestakov	Categorized as a “lifestyle” app on Apple App Store. Features include alcohol calendar to track and personal statistics of alcohol use tracked in the app such as number of ounces and cost of alcohol consumed.
Celebrate Recovery	>100	Android; iOS	Bobbi McWilliams	Categorized as a “lifestyle” app on Google Play and Apple App Stores. Features include Celebrate Recovery meeting locator, Celebrate Recovery tools, and other resources.
Construction Industry Helpline	7	Android; iOS	Construction Industry Solutions Limited	Categorized as a “health & fitness” app on Google Play Store and as a “lifestyle” app on Apple App Store. Features include coping tools, mood-tracking features, resource locator, and psychoeducation.
DrinkCoach	51	Android; iOS	Humankind	Categorized as a “health & fitness” app on Google Play and Apple App Stores. Features include alcohol tracking, calorie and cost tracking, goal-setting features, and coping skill videos.
SayingWhen	56	Android; iOS	Centre for Addiction and Mental Health (CAMH), Education	Categorized as a “health & fitness” app on Google Play and a “medical” app on Apple App Store. Features include alcohol use tracking, urge tracking, coping skills, and goal setting.
SMART Recovery	35	Android; iOS	SMART Recovery USA	Categorized as a “lifestyle” app on Google Play and as a “health & fitness” app on the Apple App Store. Features include SMART Recovery meeting locator, coping tools, and psychoeducation.
TryDry	>100	Android; iOS	Alcohol Change UK	Categorized as a “health & fitness” app on Google Play and Apple App Stores. Features include alcohol use tracking, coping skills, goal setting, and “badges” for reinforcement of achievements logged in the app.
VetChange	14	Android; iOS	US Department of Veterans Affairs	Categorized as a “health & fitness” app on Google Play and Apple App Stores. Features include alcohol tracking, coping skills, mental health tools, and goal setting.

### Themes From User Reviews

Reviews varied in length and each new idea or utterance was given a unique code for a total of 1353 coded phrases. Initially, 14 themes were identified but codes that contained <1% of the total data were collapsed into other, broader codes. Specifically, the theme of technological distrust was collapsed into “criticism” and the themes of “credibility” and “nonjudgmental” were collapsed into “praise.” The final codebook contained 11 codes, listed in Table 2.

**Table 2.** Rank-ordered list of thematic codes and their frequencies.

Rank	Theme	Examples	Frequency	%
1	Praise	“Absolutely fantastic,” “Definitely a useful little app,” “This app helped me just raise my awareness of how much I was drinking”	498	36.831
2	Tools	“Useful links to pdfs, “lots of good tools”	150	11.062
3	Suggestion	“Being able to create my own drink category, for example Hard Seltzer,” “it doesn’t allow me to manually input drink log data for any date prior to the current month. Please [sic] fix this!”	118	8.756
4	Criticism	“It’s not very flashy or feature rich,” “Terrible interface.”	105	7.768
5	Tracking	“Keeps you accountable by having you log how many drinks you have to keep up with daily,” “it helps keep track of dry days, mood, energy levels and alcohol cravings”	104	7.724
6	Personal Story	“HI!! I’M GRATEFUL BELIEVER IN JESUS WHO use to struggle with alcohol & Denial,” “I personally didn’t get any benefit from trying to put my recovery in the hands of a ‘higher power’, but I don’t mind that some people do.”	86	6.366
7	Technical Issue	“It also erased data from two days ago,” “but it it [sic] quit working after the ios update that just came out”	79	5.808
8	Usability	“Easy to use, beautifully presented and functional app,” “really easy to use”	77	5.697
9	Null	“U r funny,” “I Cannot Cannot Get Pads Your Bernhardt”	66	4.885
10	Advice	“Get Help!,” “If you want to recover you’ve got to come out of isolation.”	38	2.812
11	Gamification	“There are badges to claim,” “I love getting entering [sic] ‘stayed dry’ in the calendar and seeing the confetti. The reward really works!”	31	2.291

### Top 5 Themes

The top 5 most commonly occurring themes were praise, tools, suggestions for improvement, criticism, and tracking (see Table 2 for a rank-ordered list of all the themes coded and their frequencies). Within the theme of “praise,” reviews frequently contained general praise for the app such as “absolutely fantastic” but reviews within this category also frequently mentioned specific, positive outcomes they attributed to the app. For instance, many reviews contained concrete drinking outcome data as well as other concrete outcomes they experienced: “52 days not drinking today and soon 4 month not smoking too!!!!!,” “No doubt sleeping better,” and “Has massively helped to cut down!”

Closely related to “praise” was the second most frequently coded theme of “tools,” which was used to identify reviews that spoke specifically to functional tools programmed into the app that were not just tracking tools (see “tracking” code descriptions in the final paragraph of this section). To better inform future app development and refinement, any time “tools” were coded in the present analyses, the authors noted which specific tools were referenced as helpful by the app users. Future app programming efforts are recommended to consider these tools in their apps. Example tools that were commonly mentioned as being enjoyed and helpful were time- and location-specific reminders and specific coping tools such as guided mindfulness exercises and ways of coping with cravings, goal-setting functions, community resources, and psychoeducational resources. Examples of reviews that contained “tools” content are “useful links to pdfs and videos which are supplementary,” “There’s access to podcasts and videos, local meetings, motivational memes,” and “gives you info on where you are compared to city wide average drinkers.”

Similar to the “tools” code, the code for “suggestions for improvement” was used to indicate when users desired tools or features of the app that did not exist in the current version of the app being used. The authors also noted which specific suggestions were requested to best inform future app development efforts. The most common suggestion for improvement in apps was related to personalization and flexibility in the tracking components of the app. For instance, several instances of “suggestion for improvement” were coded for TryDry in relation to US users requesting US-specific quantities and standard drinks (ounces, 14-g standard drinks) since the app was developed in the United Kingdom (milliliters, 8-g standard drink units). One user of the TryDry app said in their review: “The one thing that could be better is using oz for the US but I understand it is for UK.” Other app reviews for TryDry and the other apps analyzed in this study called for personalization and flexibility around entering their drink information and having the app calculate standard drinks for them. Examples of this suggestion include “Have a calculator to convert units to abv% ,” and “Really don’t understand why it can’t show half units on the calendar.” Other suggestions for improvement included increased tracking features such as the ability to add notes to document particular circumstances that may explain their tracked event (eg, specific events that related to increased drinking and different moods), the abilities to customize the display settings on the tracking calendar (eg, start the week on a Monday vs a Sunday), and the option for daily reminders or notifications to ensure that users accurately tracked their drinking (eg, several apps automatically assumed that a day without alcohol consumption entered was an alcohol-free day, and several users mentioned that this led to inaccurate tracking during times when they forgot to enter drinks consumed, so a daily notification to confirm lack of alcohol consumption would be helpful instead).

It is intuitive that one may take the time to write reviews only for apps about which they have strong opinions; therefore, it is unsurprising that “criticism” was one of the top 5 most frequently coded themes. In contrast to “suggestions for improvement,” reviews that contained utterances coded as “criticism” did not provide specific, constructive feedback. As such, the authors did not note specific subthemes within this category and the majority of reviews coded with “criticism” did not yield particularly helpful feedback for future app development or improvement. Examples of reviews that contained “criticism” included “Having 1 drink in the past 30 days means I am drinking more than “28%” what bunk statistics is this based on. It acts like I’m abusing substances because I had one drink,” “I really wish I could rate this better, but it just doesn’t work at all,” and “Very frustrating.”

Nearly equal to the frequency with which “criticism” was coded was the code for “tracking” in the present analyses. While potentially considered a “tool,” the mention of “tracking” specifically was so common that the authors agreed that it warranted its own code. Examples of reviews containing “tracking” codes were “keeps you accountable by having you log how many drinks you have to keep up with daily; and weekly goals,” “Good tool to track drinks per day,” and “has helped me a lot to track my drinking habits.” However, tracking capabilities across the reviewed apps were not limited solely to tracking drinking behavior, and several reviews specifically mentioned the helpfulness of tracking more than drinking behavior alone. The most commonly mentioned tracking features that were mentioned as helpful to track included cost, calories, cravings, and mood. That “tracking” was among the top 5 most frequently mentioned codes beyond “tools” more broadly highlights just how valuable and well-liked tracking features are in alcohol apps.

### Additional Themes

The remaining 6 codes were observed in moderate and low frequency, compared with the codes of the top 5. “Personal story,” “technical issues,” “useability,” and “null” codes were found in moderate frequency, comprising around 5% of the total codes each. The “personal story” and “null” codes were used when the review contained information that was not clearly related to the app. For instance, many user reviews contained personal anecdotes about their lives that may have led them to seek help through the mobile app but did not indicate any information for others about the app per se (eg, “Other 12 step programs weren’t doing it for me.”). The “null” code was used for reviews that were non-English or contained nonsensical text (eg, “I Cannot Cannot Get Pads Your Bernhardt”) or where the text clearly was reviewing something outside of the app itself such as reviews of in-person meetings associated with but not part of the app directly (ie, SMART Recovery or Celebrate Recovery in-person meetings). Complaints about particular “technical issues” or glitches experienced in the apps were also found at moderate frequency, likely highlighting a need for apps to have tech support options available. Conversely, “useability” was coded when reviews mentioned positive qualities about the user experience from an app interface perspective.

At lower but still prominent frequencies were the codes for “advice” and “gamification.” While less useful feedback for mobile app development and refinement, many reviews contained advice to others that may or may not have been explicitly about the app itself. The “advice” code was found for most but not all of the apps reviewed and so may speak to population-level differences (eg, veterans advocating for other veterans and in-person meeting-focused apps advocating for community). For TryDry and DrinkCoach, several reviews mentioned the gamification that has been programmed to reinforce abstinence days such as confetti appearing on the screen to celebrate abstinent day entry. Other app reviewers also highlighted that color coding of drinking data tracking that aligns with their drinking goals was helpful. The fact that only 2 of the 8 total apps evaluated in this study contained mention of gamification and yet still was coded 31 times speaks to the high desirability of gamified features for mobile apps and suggests that other apps should program gamified functionality.

## Discussion

### Principal Results

Central to the primary aims of the study, qualitative analyses yielded significant insights into features of alcohol-focused mobile apps that may inform app development and enhancement to make apps more accessible and helpful. Broadly, the central research question of this project was to identify overarching themes across alcohol app user reviews. We found common topics of reviews shown in Table 2. However, while conducting formal qualitative analyses of these overarching themes, both individual coders also took note of any mention of specific features that were praised, suggested, or criticized that may provide deeper insights for future app developers. Given these were subthemes and counts were inherently very small compared with the overall themes identified in Table 2, formal qualitative analyses were not completed to obtain counts of each specific feature highlighted by reviewers. Nevertheless, these subthemes may be particularly helpful for guiding future app development and enhancement, given their specificity. Features that were commented in user reviews as helpful or desired include tools to help access resources (eg, in-person meeting locators and psychoeducational materials) or coping skills, customizability to add personalized notes, and tracking functionality. While several user reviews commented on the helpfulness and importance of tracking alcohol use itself, being able to calculate standard drinks flexibly and track nondrinking data such as mood, craving, costs, and calories was prominent across mobile app reviews. The presence of reminder capabilities was also frequently commented on as a helpful tool to enhance the benefits of tracking features of apps. Moreover, including gamification (eg, color changes of tracking data when goals are met and rewarding displays such as confetti for achieving goals) explicitly into app programming was well received by app users (gamification was coded 31 times despite the majority of the reviewed apps lacking gamification features). Of note, only 2 of the 8 apps included in this study contained user reviews of “gamification” codes, yet “gamification” alone consisted of 2.29% of all coded responses. If gamification was programmed into more of the apps, one would expect that the proportion of reviews mentioning “gamification” would have increased significantly.

### Additional Insights on mHealth Mobile Apps

A number of mHealth mobile apps are available for individuals interested in changing their relationship with alcohol. On one hand, the ubiquity of mobile apps provides individuals with many options to access resources that may not otherwise be available to them. On the other hand, without research and regulations, serious negative consequences can occur if people find the apps unhelpful or harmful [11]. In this study, we reviewed freely available mobile apps that may help individuals reduce their alcohol use and alcohol-related harms. We found hundreds of results, yet the vast majority of these mobile apps were available for-profit, for entertainment and potential harm (eg, drinking game apps), or for single purposes that restrict the potential usefulness of an app (eg, blood alcohol concentration calculators). This information alone highlights one current problem for the state of app development.

In addition to the current potential harms for app stores providing such limited mHealth interventions as opposed to predatory products, another important finding from this study is the inherent limitations in app store search features. For instance, Apple App yielded drastically different search results compared with Google Play. Moreover, apps known to exist failed to be returned in search results (eg, DrinkLess and DrinksRation from the United Kingdom, and Less, which is currently available on iPhone in the United States). Given the present methodology for searching not only for “alcohol” but also for other highly related terms (ie, “alcohol and anxiety”), this finding is concerning. One of the primary advantages of mobile apps is their accessibility; however, if the majority of available apps are for-profit or not viewable with very basic search terms, many individuals who may benefit from mHealth interventions may fail to access them.

One anticipated theme for this study was that privacy concerns may play a large role in user reviews, given concerns regarding privacy and mHealth in the academic literature [17]. However, this concern did not feature prominently in user reviews, with only 1 review mentioning concerns regarding location tracking in the app. This suggests that these users did not find privacy concerns to be a barrier to mHealth app adoption. However, it also highlights that mHealth app users may not be attending to their data privacy, and thus it is important for mHealth apps to provide transparent data usage policies and follow best practices regarding maintaining mHealth data [18]. Other primary takeaways from the user reviews include areas of criticism that highlight the need for improvements in existing apps (criticism was coded 105 times). Specifically, user interface, the ability for the app to perform multiple tasks (ie, not simply a meeting locator and readings interface), availability of technical support to report glitches with the app, and customizability were highly sought (eg, ability to change volume measurements from milliliters to ounces). Future app developers and organizations with active app portfolios are recommended to use these findings to develop more well-received apps.

### Limitations

While many of the present findings have significant and novel implications, the study is not without limitations. One limitation is that not all known alcohol-related apps were included in the present analyses. However, this is useful information since it highlights the inadequacies of current search engines in mobile app stores and shows a current gap that needs to be addressed by app developers and app stores in order for their products to be useful to real-world users. A second limitation is that not all existing app reviews were examined in this study. Specifically, TryDry and Celebrate Recovery had several hundred of reviews across Google and Apple but the present analyses were restricted to only the most recent 50 reviews from each store (100 reviews total for each app). Accordingly, additional nuances may have been missed that would have resulted in different codes in our qualitative analyses. However, had all reviews been included, the current findings would not generalize to other alcohol-related apps since the codes would be overly specific to those 2 apps alone. The fact that “gamification” occurred frequently enough to warrant a code and, yet, it was only a feature in 2 of the 8 apps highlights the appeal of gamified features within apps to users. Furthermore, many “technical issues” that were found may have been specific to unique versions of an app, so analyzing all existing reviews may have resulted in skewed findings about glitches or other app features that no longer exist in the current apps. Another limitation of this study was the restriction to English language apps. In our search for freely available mobile apps, we did find non-English apps such as “Oz Ensemble” developed by Fabrique numérique des ministères sociaux. The inclusion of non-English apps may have resulted in different findings, and future studies should be done to examine and compare non-English apps with the present results. Another point of potential limitation in our findings was the reliance on limited tools to examine the mobile app stores more systematically such as machine learning or other techniques that require different expertise or software support. Given the present research was completed without independent funding, our methodology was restricted to approaches that were freely available and fell within the scope of our existing expertise; it is possible that purchasable or other tools that conduct systematic reviews of app stores may have yielded different findings. A final limitation of this study is the inherent limitations of using publicly available user review data as opposed to other methodology for app development such as UX studies [19]. Yet, the advantages of UX studies may also be considered disadvantages that the present methodology addresses: UX studies are costly and formal. The current data were obtained freely and without the same potential desirability that may result from formal research, which may have resulted in different findings than if this study had been conducted through formal research methods such as UX studies [19].

#### Comparison to Prior Work

The present findings echo previous research regarding mHealth interventions for substance use. Specifically, that “nearly all of the mHealth interventions that are available for download today have not been evaluated for efficacy or effectiveness” [7]. In addition, the wide difference in the number of available reviews across apps evaluated in this study highlights findings from previous research that user’s ratings influence perceptions about the apps’ overall quality [7]. TryDry and Celebrate Recovery had significantly more user ratings and reviews than the other apps, and those apps have more downloads. Moreover, our findings are consistent with previous research examining app user reviews of nonsubstance use mental health apps, which highlight user preferences for usability, minimization of technical issues, and personalization [20-22]. As such, app developers should listen to what user reviews say in order to learn what makes one app successful compared with another from user experience and access perspectives. Also consistent with previous studies of formal AUD treatment and behavior change literature more broadly, skills-based tools and tracking capabilities of alcohol-related mobile apps arose as 2 of the most widely referenced content in the present qualitative analyses.

### Conclusions

Mobile apps hold great promise to help the millions of individuals worldwide who experience serious harms associated with alcohol use. They may reach a wider audience than traditional, in-person therapy due to the ubiquity of smartphones and the free nature of these apps. Moreover, as compared with in-person treatment, receiving help in changing one’s relationship with alcohol through a mobile app provides the app user with inherently increased privacy and autonomy. However, many apps that have been developed are not accessed or are not science-based: this study highlights ways to translate evidence-based techniques to mobile apps by listening to what the app users themselves have to say. Although many of the reviews for these apps are similar to reviews of any product by being either generic praise or generic, nonconstructive criticism, many of the present findings highlight concrete features that users want in an app to help change their relationship to alcohol. Flexible and versatile tools to access resources such as coping skills, psychoeducation, and community support; easy to use and rewarding or fun tracking capabilities; and ways for users to individualize their experience stand out among the more than 1300 coded utterances contained in the reviews that were examined. Similar to traditional therapy, skills-based and behaviorally based interventions that allow users autonomy were widely recognized as being helpful, appreciated, or, where lacking in an app, desired.

## Supplementary material

10.2196/63148 Checklist 1 PRISMA checklist.

## Abbreviations

ADHD: attention-deficit/hyperactivity disorder
AUD: alcohol use disorder
mHealth: mobile health
PTSD: posttraumatic stress disorder
UX: User Experience
